# Equality of specialist orthodontic care for adolescents in the Swedish public dental service: a cohort study

**DOI:** 10.1186/s12903-025-06220-x

**Published:** 2025-05-28

**Authors:** Emma Göranson, Mikael Sonesson, Lillemor Dimberg, Niko Vähäsarja, Aron Naimi-Akbar

**Affiliations:** 1 Center for Orthodontics and Pediatric Dentistry, Norrköping, County Council of Östergötland, 58186 Linköping, Sweden; 2 Department of Orthodontics, Faculty of Odontology, Malmö University, Malmö, Sweden; 3 Swedish Agency for Health Technology Assessment and Assessment of Social Services, Stockholm, Sweden; 4 Department of Clinical Sciences Malmö, Lund University, Malmö, Sweden; 5 Department of Dental Medicine, Karolinska Institutet, Stockholm, Sweden; 6 Health Technology Assessment Odontology (HTA-O), Faculty of Odontology, Malmö University, Malmö, Sweden

**Keywords:** Cohort study, Orthodontics, Sociodemographic Factors, Healthcare disparities

## Abstract

**Background:**

In Sweden, dental care for children and adolescents, including specialist orthodontic treatment, is publicly funded. This study aims to analyze the impact of sociodemographic factors on the distribution of publicly funded specialist orthodontic treatment in a mid-sized Swedish region.

**Methods:**

A registry-based cohort study was conducted in Region Östergötland, including individuals born between 2000 and 2003. Sociodemographic data were obtained from Statistics Sweden (SCB), while dental health information was sourced from The Swedish Quality Registry for Caries and Periodontal Disease (SKaPa). The primary outcome variable was initiation of specialist orthodontic treatment, extracted from dental records. Statistical analysis was performed using Stata v.18.1.

**Results:**

The cohort comprised 16 893 individuals, with 51.5% males and 48.5% females. Specialist orthodontic treatment was initiated for 25.7% of the population (*n* = 4 342), with most treatments involving fixed appliances. Several sociodemographic factors were significantly associated with the likelihood of receiving orthodontic treatment. Females had 1.74 times higher odds (95% CI: 1.63–1.87) of receiving treatment compared to males. Individuals born in Sweden had 1.42 times greater odds (95% CI: 1.18–1.72) of receiving treatment compared to those born abroad. Similarly, children with Swedish-born parents had 1.16 times increased odds (95% CI: 1.04–1.30) compared to children with foreign-born parents. Children of mothers with university/college education had an OR of 1.29 (95% CI: 1.12–1.48), while those whose fathers had a university/college education had an OR of 1.19 (95% CI: 1.05–1.34), compared to parents with primary/lower secondary education.

**Conclusions:**

Males, individuals born outside Sweden, those with foreign born parents, and whose parents had lower educational levels were less likely to receive orthodontic treatment within the publicly funded dental health services in Region Östergötland. These findings suggest that sociodemographic factors influenced the distribution of orthodontic care, though the role of treatment demand requires further investigation.

**Supplementary Information:**

The online version contains supplementary material available at 10.1186/s12903-025-06220-x.

## Background

The Swedish healthcare system is designed to provide equitable care to all individuals, with a focus on prioritizing those with the greatest need [1]. However, significant disparities in health outcomes and access to healthcare have been observed across different Swedish regions, between sexes, and among socioeconomic groups [2].

Similar to the health care system, the Swedish dental healthcare system aims to provide *“good dental health and dental care on equal terms for the entire population*” [3].

Unfortunately, the Swedish dental healthcare system for adults is unequal. A public investigation published in 2021 highlighted significant disparities [4]. The frequency of dental visits is lower in sparsely populated counties as well as in socioeconomically vulnerable metropolitan areas. Additionally, young women visit dental examinations more often than expected given their dental health. Furthermore, foreign-born individuals are three times more likely to have unmet dental care needs than individuals born in Sweden [4]. A greater risk of underserved oral care needs has similarly been reported among those with low income [5, 6]. One contributing factor to these inequalities may be the financing structure of adult dental care, which relies heavily on out-of-pocket payments with only limited public subsidies.

For Swedish children and adolescents, dental care is publicly funded and provided free of charge. Both public dental services and private practitioners deliver publicly funded general dental care for children and adolescents. Specialist orthodontic treatment is available at no cost for those deemed in need; however, in most of the 21 Swedish regions, all orthodontic patients are referred to public dental services for treatment. Approximately 25–30% of Swedish children and adolescents receive publicly funded specialist orthodontic treatment at some point during their childhood or adolescence [7]. There is no national regulation or guideline specifying which malocclusions should be treated. Instead, each region sets its own criteria.

Despite the provision of free dental care for children and adolescents, disparities in dental health and access to care persist. Caries is most prevalent among children whose parents are foreign-born, have low incomes, and/or with lower levels of maternal education [8–12]. Furthermore, children of lower socioeconomic status and an immigrant background more often fail to appear at dental visits and therefore miss out on treatment [13].

Low caries risk and good oral hygiene are prerequisites for orthodontic treatment, since placing orthodontic braces on teeth inhibits oral hygiene and increases the risk of severe dental decay [14]. Consequently, individuals from lower socioeconomic backgrounds and those with immigrant background may face barriers to accessing the specialist orthodontic care they need [15–18].

Internationally, the funding models for specialist orthodontic treatment for children and adolescents differ significantly. Research on privately funded specialist orthodontic care schemes has often revealed disparities in treatment distribution [16, 19, 20], which is perhaps unsurprising. However, dissimilarities may exist even in publicly funded dental healthcare systems. A recent study from Norway indicated an equitable distribution of specialist orthodontic care within their publicly funded system [21]. In contrast, the British NHS, despite being publicly funded, has shown notable inequalities in treatment distribution [15, 18, 22–25]. Similarly, a German study on orthodontic treatments, which are fully covered or reimbursed by public health insurance, revealed comparable disparities [20]. The existence of disparities even within publicly funded dental healthcare systems highlights the importance of investigating this issue further.

Potential sociodemographic differences in publicly funded specialist orthodontic care in Sweden have not yet been explored but merit further investigation. Sweden offers a unique opportunity to study sociodemographic differences in distribution of specialist orthodontic care. The country’s high-quality registries on dental health, dental care, and sociodemographic variables provide comprehensive data, minimizing the risk of bias and enhancing the reliability of findings. In contrast, many previous international studies have used less reliable self-reported measures of orthodontic treatment and/or socioeconomic status [15–19]. Furthermore, mapping potential sociodemographic differences in specialist orthodontic care is especially important in a country such as Sweden, where treatments are publicly funded.

The aim of the study is to investigate the impact of sociodemographic variables on publicly funded specialist orthodontic treatment in a mid-sized Swedish region. By examining the distribution of specialist orthodontic care, the study intends to provide valuable scientific insights that benefit both society and young prospective patients.

## Methods

### Setting

The study was conducted in Östergötland, a mid-sized region in southeastern Sweden with a population of 465 215 inhabitants as of November 1, 2019. In Östergötland, as in most parts of Sweden, both public dental services and private practitioners provide publicly funded general dental care for children and adolescents. When a general practitioner identifies a potential need for orthodontic treatment, the patient is referred for a consultation with a specialist orthodontist. If a need for treatment is confirmed, publicly funded specialist orthodontic care in Region Östergötland is provided exclusively by the public dental services. At the time of data collection for this study, specialist orthodontists in Region Östergötland used two different orthodontic treatment indices to assess patient eligibility for treatment. Patients were offered treatment if they had an ICON index of ≥ 44 and/or demonstrated a treatment need based on a modified version of the IOTN index [26, 27].

### Study design

This registry-based cohort study obtained ethical approval from The Swedish Ethical Review Authority (Dnr 2020/03628). The study follows the RECORD Statement checklist of items that should be reported in observational studies using routinely collected health data [28].

The cohort included all individuals born between 2000 and 2003 residing in Region Östergötland between the ages of 8 and 15 years. The cohort was identified by Statistics Sweden (SCB).

The primary exposure variables consisted of sociodemographic data on the cohort and their parents, obtained from SCB. Secondary exposure variables were dental health data on the cohort, obtained from The Swedish Quality Registry for caries and periodontal disease (SKaPa). The outcome variable was specialist orthodontic treatment, with the primary outcome being the initiation of treatment. This information, along with details on the type and length of treatment, was retrieved from the electronic dental records of Region Östergötland. SCB linked individuals across the registries using the personal identification number (PIN) [29]. The datasets were anonymized and assigned constructed identification numbers before delivery to the research group.

**Fig. 1:**
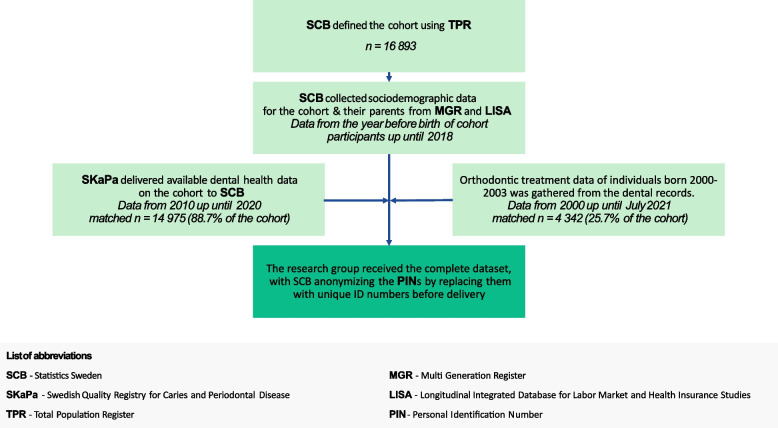
Flow chart depicting data sources and the workflow

The data sources and the work flow are presented in Fig. 1.

### Primary exposure—Sociodemographic variables

Parental data were collected from the biological mother and father, or, when applicable, replaced by adoptive parents. Data from the year of birth of the cohort participant up until 2018 (when adolescents were aged 15–18 years) was available and included. Table 1 presents all exposure variables that were analyzed in the study, while Supplementary Table 1 provides a detailed description of each variable and the data cleaning procedure.

**Table 1:** Exposure variables potentially affecting the outcome variable (specialist orthodontic treatment)

	***n***	**% of total cohort (16 893)**
**Main exposure—Sociodemographic variables**		
**Sex**		
Male	8 691	51.5
Female	8 202	48.5
**Year of birth**		
2003	4 417	26.1
2002	4 323	25.6
2001	4 022	23.8
2000	4 131	24.5
**Country of birth**		
Foreign born	1 054	6.2
Swedish born	15 839	93.8
Parental country of birth		
Both parents foreign born	2 068	12.2
One parent foreign born	1 455	8.6
Both parents Swedish born	13 039	77.2
*Data unavailable*	*331*	*2.0*
**Mother’s DEGURBA**^**†**^		
Thinly populated area	2 777	16.4
Intermediate density area	3 252	19.3
Densely populated area	10 700	63.3
Outside Östergötland	74	0.4
*Data unavailable*	*90*	*0.5*
**Father’s DEGURBA**^**†**^		
Thinly populated area	2 676	15.8
Intermediate density area	3 158	18.7
Densely populated area	10 182	60.3
Outside Östergötland	377	2.2
*Data unavailable*	*500*	*3.0*
**Parental living arrangement**^**‡**^		
Living separately	3 404	20.2
Living together	12 263	72.6
*Data unavailable*	*1 226*	*7.3*
**Mother’s educational level**^**‡**^		
Primary/lower secondary school	1 631	9.7
Upper secondary school	7 719	45.7
University/college education	7 420	43.9
*Data unavailable*	*123*	*0.7*
**Father’s educational level**^**‡**^		
Primary/lower secondary school	1 985	11.8
Upper secondary school	8 867	52.5
University/college education	5 684	33.7
*Data unavailable*	*357*	*2.1*
**Parental social welfare**^**§**^		
Both parents with social welfare	1 861	11.0
One parent with social welfare	947	5.6
No social welfare	14 085	83.4
**Parental unemployment**^**¶**^		
Both parents unemployed	998	5.9
One parent unemployed	3 965	23.5
No unemployment	11 920	70.6
**Parental income**^**#**^		
1 st (lowest) quintile	3 374	20.0
2nd quintile	3 375	20.0
3rd quintile	3 375	20.0
4 th quintile	3 382	20.0
5 th (highest) quintile	3 378	20.0
*Data unavailable*	*9*	*0.0*
**Secondary Exposure—Dental health variables**		
**DFT primary at age 10**		
≥ 5	1 519	9.0
1–4	4 498	26.7
0	8 619	51.0
*Data unavailable*	*2 257*	*13.4*
**DFT permanent at age 14**		
≥ 5	950	86.7
1–4	5 957	35.3
0	7 739	45.8
*Data unavailable*	*2 247*	*13.3*

^†^DEGURBA = degree of urbanization. At cohort participant aged 10 years

^‡^At cohort participant aged 10 years

^§^Parental social welfare at any time during the cohort participant’s childhood (when aged 0–10 years)

^¶^Parental unemployment for six months or more at any time during the cohort participant’s childhood (when aged 0–10 years)

^#^Net yearly income, mean of mother and father, adjusted for number of family members, quintiles

### Secondary exposure—Dental health variables

Data from SKaPa included dental health data from 2010–2020 for study participants who visited clinics affiliated with SKaPa. Table 1 presents all exposure variables analyzed in the study, while Supplementary Table 1 provides a detailed description of each variable and the data cleaning procedure.

### Outcome variable—Specialist orthodontic treatment

Specialist orthodontic treatment data were extracted from the orthodontic dental records of the public dental services of Östergötland (*Edward 32, Pro Curis Inc*.) in collaboration with the support team. Data retrieval was conducted using an SQL query to ensure accurate and systematic extraction. Data were available and included from the year 2000 (corresponding to the birth year of the first born adolescents in the cohort) until July 2021, when the adolescents were aged 18–21 years. The dataset included information on the start date of treatment, type of treatment (removable or fixed), and treatment completion date. The primary outcome variable was initiation of specialist orthodontic treatment. Supplementary Table 1 describes each variable and the data cleaning procedure in detail.

### Study size

In a German observational study, a greater likelihood of receiving orthodontic treatment was shown for individuals from an area with higher socioeconomic status (West Germany) than for those from an area with lower socioeconomic status (East Germany). The odds ratio for this association was 1.45 [19]. Assuming a similar increased likelihood in the higher socioeconomic status group and that 25% receive treatment in the lower socioeconomic status group in our study, with a power of 0.9 and a significance level of 0.05, we would need 747 participants in each group (totaling 1 494 individuals). Since approximately 5000 participants could be included in each birth cohort in the current region and we planned to include four birth cohorts (approximately 20 000 individuals), of which slightly more than 25% were likely to be treated (approximately 5 000 individuals), that sample size would allow us to detect much more modest differences between the groups.

### Statistics

All analyses were conducted using Stata v.18.1 software (Stata Corporation LLC, College Station, USA).

Categorical outcome variables are presented as numbers and proportions as percentages for the different exposure groups. Continuous and discrete variables are presented as the means with 95% confidence intervals (CIs) and standard deviations.

Independent samples t tests and chi-square test were used to compare differences between the groups (males and females) in orthodontic treatment data.

To guide the selection of variables for inclusion in the logistic regression analysis and adjustment for confounding, we constructed a Directed Acyclic Graph (DAG) for each of the primary exposure variables based on existing literature and subject-matter expertise [30, 31].

**Fig. 2:**
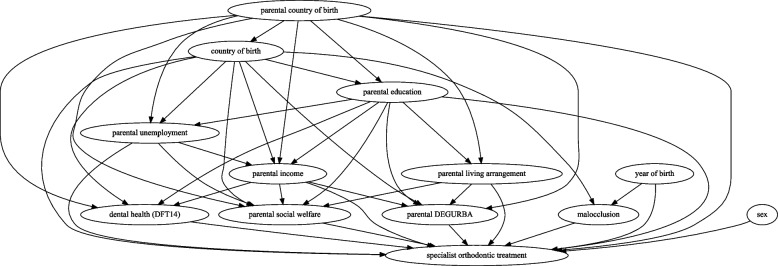
Directed Acyclic Graph depicting the causal relationship between exposure variables (sociodemographic and dental health variables) and the primary outcome variable (initiation of specialist orthodontic care). Arrows represent hypothetical causal effects. The direction of the arrows represent the presumed influence from one variable to another

The DAG was developed using DAGitty (version 3.1). A visual representation of the DAG is provided in Fig. 2. The final adjustment sets for each exposure variable was determined using DAGitty’s adjustment sets function. To avoid multicollinearity, variables related to the dental health of primary teeth were excluded, whereas those related to permanent teeth were retained.

*P* values less than 0.05 were considered statistically significant.

## Results

### Participants

The characteristics of the cohort are presented in Table 1. The cohort included 16 893 individuals born between 2000 and 2003. Among these, 51.5% were males, and 48.5% were females.

### Dental health

Dental health data were available in the SKaPa register for 14,975 individuals, representing 88.6% of the cohort. Among the cohort, 51.8% were free of caries and fillings (DFT = 0) at age 10 and 45.8% at age 14. At age 10, males had a significantly greater rate of primary tooth caries (1.20, SD 2.1) than females (1.09, SD 1.95) (*p* < 0.01). However, by age 14, no significant differences in permanent tooth caries were detected between males (1.20, SD 1.89) and females (1.27, SD 1.96) (*p* = 0.96).

### Specialist orthodontic treatment

Specialist orthodontic treatment was received by 25.7% of the population (*n* = 4 342). The vast majority of participants (*n* = 4 293, 98.9%) received treatment with fixed appliances, either alone or in combination with removable appliance, whereas only 49 individuals (0.3%) were treated solely with removable appliances (Table 2). The average age at the start of treatment was 14.6 years (SD 2.1), and the mean duration of treatment was 26.8 months (SD 11.5). Compared with males, females tended to begin treatment slightly earlier and had marginally shorter treatment durations (Table 3).

**Table 2:** Descriptive data on the outcome variable (specialist orthodontic treatment) and the difference between males and females (chi2 test)

	**Total cohort (%)**	**Males (%)**	**Females (%)**	***p*-value**
**Orthodontic treatment**				
Yes	4 342 (25.7)	1 789 (20.6)	2 553 (31.1)	≤ 0.01**
No	12 551 (74.3)	6 902 (79.4)	5 649 (68.9)	≤ 0.01**
**Treatment type**				
Fixed only or fixed with removable	4 293 (25.4)	1 759 (20.2)	2 534 (30.9)	≤ 0.01**
Removable only	49 (0.3)	30 (0.4)	19 (0.2)	0.17

^*^ *p* ≤ 0.05

^**^p ≤ 0.01

**Table 3:** Age at specialist orthodontic treatment start and orthodontic treatment duration. Independent samples T-test

	**Treated males**		**Treated females**				
	**mean**	**SD**	**mean**	**SD**	**df**	**t**	**p**
**Age at treatment start**	14.7	2.2	14.6	2.0	4340	1.8802	≤ 0.05*
**Treatment duration** (months)	27.2	12.0	26.6	11.2	3571	1.6986	≤ 0.05*

^*^ *p* ≤ 0.05

### Effect of sociodemographic variables on specialist orthodontic treatment

The effect of each sociodemographic variable on specialist orthodontic treatment, along with the final adjustment sets for each exposure variable, is presented in Table 4. Several sociodemographic factors were significantly associated with the initiation of specialist orthodontic treatment. Females had a higher odds ratio (OR = 1.74, 95% CI: 1.63–1.87) compared to males. An earlier year of birth was also associated with increased odds of treatment initiation, with the OR ranging from 1.24 (95% CI: 1.12–1.37) for those born in 2002 to 1.52 (95% CI: 1.38–1.68) for those born in 2000, compared to individuals born in 2003.

**Table 4:** Exposure variables effect on the primary outcome variable (initiation of specialist orthodontic treatment). Simple and multivariable logistic regression

	OR	95% CI lower	95% CI higher
**Main exposure—Sociodemographic variables**			
Sex^*^			
Male	1	1	1
Female	1.74	1.63	1.87
Year of birth^*^			
2003	1	1	1
2002	1.24	1.12	1.37
2001	1.36	1.23	1.50
2000	1.52	1.38	1.68
**Country of birth**^**†**^			
Foreign born	1	1	1
Swedish born	1.42	1.18	1.72
Parental country of birth^*^			
Both parents foreign born	1	1	1
One parent foreign born	1.17	1.00	1.36
Both parents Swedish born	1.16	1.04	1.30
**Mother’s DEGURBA**^§^			
Thinly populated area	1	1	1
Intermediate density area	1.13	1.00	1.27
Densely populated area	1.07	0.96	1.18
Outside Östergötland	0.96	0.48	1.89
**Father’s DEGURBA**^§^			
Thinly populated area	1	1	1
Intermediate density area	1.11	0.98	1.25
Densely populated area	1.06	0.95	1.17
Outside Östergötland	1.01	0.75	1.36
**Parental living arrangement**^**¶**^			
Living separately	1	1	1
Living together	1.02	0.93	1.11
**Mother’s educational level**^#^			
Primary/lower secondary school	1	1	1
Upper secondary school	1.20	1.05	1.38
University/college education	1.29	1.12	1.48
**Father’s educational level**^**#**^			
Primary/lower secondary school	1	1	1
Upper secondary school	1.12	0.99	1.26
University/college education	1.19	1.05	1.34
Parental social welfare**			
Both parents with social welfare	1	1	1
One parent with social welfare	1.12	0.90	1.40
No social welfare	1.17	1.01	1.35
**Parental unemployment**^††^			
Both parents unemployed	1	1	1
One parent unemployed	0.94	0.80	1.11
No unemployment	1.03	0.88	1.21
**Parental income**^‡‡^			
1 st (lowest) quintile	1	1	1
2nd quintile	1.07	0.94	1.21
3rd quintile	1.03	0.91	1.18
4 th quintile	1.09	0.95	1.24
5 th (highest) quintile	1.19	1.04	1.36
**Secondary Exposure—Dental health variables**			
**DFT permanent at age 14**^§§^			
≥ 5	1	1	1
1–4	1.38	1.15	1.66
0	1.42	1.18	1.71

^*^Simple logistic regression

^†^Multivariable logistic regression, adjusted for parental country of birth

^‡^Multivariable logistic regression, adjusted for year of birth

^§^Multivariable logistic regression, adjusted for country of birth, parental country of birth, parental education, parental income, and parental living arrangement

^¶^Multivariable logistic regression, adjusted for parental country of birth, and parental education

^#^Multivariable logistic regression, adjusted for country of birth, and parental country of birth

^**^Multivariable logistic regression, adjusted for country of birth, parental country of birth, parental education, parental income, parental living arrangement, and parental unemployment

^††^Multivariable logistic regression, adjusted for country of birth, parental country of birth, and parental education

^‡‡^Multivariable logistic regression, adjusted for country of birth, parental country of birth, parental education, and parental unemployment

^§§^Multivariable logistic regression, adjusted for country of birth, parental country of birth, parental education, and parental income

Individuals born in Sweden had greater odds of receiving orthodontic treatment (OR = 1.42, 95% CI: 1.18–1.72) compared to foreign-born individuals. Similarly, having both parents born in Sweden and one parent born in Sweden was associated with increased odds compared to having two foreign-born parents (OR = 1.16–1.17).

Higher parental educational attainment was associated with increased odds of treatment. Children of mothers with university/college education had an OR of 1.29 (95% CI: 1.12–1.48), while those whose fathers had a university/college education had an OR of 1.19 (95% CI: 1.05–1.34), compared to parents with primary/lower secondary education.

Parental income slightly influenced the likelihood of orthodontic treatment, with children from the highest income quintile having increased odds (OR = 1.19, 95% CI: 1.04–1.36) compared to those in the lowest quintile. However, no clear gradient was observed across quintiles, and the OR for the second, third and fourth quintiles did not show significant associations with treatment uptake.

In contrast, no statistically significant associations were found between orthodontic treatment initiation and parental living arrangement or parental unemployment. Additionally, the variables of parental social welfare and parental residential area (DEGURBA) within Region Östergötland displayed marginally significant results in some categories, though these findings were not consistent across all groups.

### Effect of dental health variables on specialist orthodontic treatment

Individuals with a DFT of 0 were more likely to receive treatment than those with a DFT ≥ 5 (OR 1.42, 95% CI 1.18–1.71) and those with DFT 1–4 (OR 1.38, 95% CI 1.15–1.66) (Table 4).

## Discussion

This study confirms that sociodemographic factors influenced the distribution of publicly funded orthodontic care in Region Östergötland, with observed differences based on sex, parental education, and country of birth. While treatment demand discrepancies may contribute to these variations, the role of structural factors, such as healthcare access and health beliefs, warrants further investigation. The findings highlight the need for policies that ensure equitable distribution orthodontic care.

Females were more likely to receive specialist orthodontic treatment than males. The sex distribution in our study closely aligns with the commonly reported distribution of orthodontic patients, with approximately 40% males and 60% females [32]. This disparity persists even though malocclusions are equally common in both sexes [33–35]. One possible explanation is that girls have a greater self-perceived need for specialist orthodontic treatment and are more dissatisfied with their teeth than boys are [36, 37]. Therefore, girls may seek orthodontic treatment to a greater extent. Since malocclusions are not a disease but rather a deviation from societal norms, it may be reasonable that those who actively seek treatment receive a greater degree of care. However, it remains a subject of debate whether public dental services should address these sex disparities in treatment—and if so, whether this would involve reducing orthodontic treatment for girls, increasing it for boys, or adopting other measures to ensure equitable distribution.

The decision to include individuals who had resided in Region Östergötland for at least the ages of 8 to 15 was based on the fact that orthodontic treatment rarely begins before the age of 8. This criterion ensured that the study captured individuals who were present during the typical age range for orthodontic assessment and treatment initiation. The selection of the four specific age cohorts was aimed at optimizing data coverage. Since data collection took place in the fall of 2019 and SkaPa data became available in 2010, we excluded individuals born before 2000 to ensure access to dental health records from age 10. Simultaneously, we aimed to include the oldest possible cohort at the time of data collection to maximize the likelihood that planned orthodontic treatments had already commenced.

As expected, older adolescents were more likely to have begun specialist orthodontic treatment. The adolescents were aged 18–21 when treatment data was collected. It is possible that a few of the younger individuals were still on waiting lists for treatment. This might be particularly relevant in the studied region, where specialist orthodontic resources are limited, and waiting times for orthodontic care have ranged from 1 to 3 years during the studied time period.

Swedish-born individuals were more likely to receive orthodontic treatment than those born abroad. It remains unclear whether this disparity reflects differences in treatment demand between the two groups or if the public dental health system does not provide treatment on equal terms. Further research is needed.

Having both parents born in Sweden or one parent born in Sweden increased the odds of receiving orthodontic treatment as compared to having two foreign-born parents. While the exact reasons for this disparity remain unclear, previous research suggests that differences in healthcare utilization among immigrants may be attributed to both organizational factors, such as access to services, and individual factors, including health beliefs. These health beliefs, particularly an individual's self-perceived need for and sense of entitlement to care, may influence the likelihood of seeking treatment [38].

Higher parental educational levels were associated with increased odds of receiving orthodontic treatment. Previous research has shown that parental educational level influences children’s oral hygiene practices and oral health [39, 40]. Additionally, higher parental educational levels may be associated with an increased likelihood of actively seeking orthodontic treatment.

As expected, individuals with no caries were more likely to receive orthodontic treatment than those with caries. In the DAGitty analyses, several of the sociodemographic variables had to be adjusted for dental health in order to close biasing paths. The higher burden of caries in lower socioeconomic groups and in immigrants is not merely a matter of individual choice but is shaped by structural inequalities and social determinants of health. These disparities highlight the need for preventive strategies aimed at improving dental health across all groups, ensuring that every child has sufficiently healthy teeth to qualify for orthodontic treatment.

This study found that several sociodemographic variables significantly influenced the likelihood of receiving publicly funded specialist orthodontic treatment in a Swedish region. This is an unwanted outcome, as it suggests that orthodontic treatments are perhaps not equitably distributed. Similarly, research reports on the publicly funded British NHS and the German insurance system indicate that parental socioeconomic status affects treatment uptake [15, 18, 20, 24, 25]. In contrast, a recent Norwegian study revealed an equitable distribution of their publicly funded specialist orthodontic treatment [21]. The reported disparities between countries may stem from differences in healthcare policies, referral systems, and the extent of patient cost-sharing, as well as variations in study design.

The research findings highlight the importance of policy design in ensuring equitable distribution of orthodontic treatment. Further research could explore the specific factors that contribute to these differences, such as referral pathways and parental awareness of treatment options. Understanding these factors could inform policy improvements to enhance equity in distribution of orthodontic treatment across different healthcare systems.

### Strengths and weaknesses

A strength of the study is the extensive coverage and accuracy of the Swedish national registers. The Swedish personal identity number is a useful tool for linkages between registers and has virtually 100% coverage [29]. The TPR and the MGR have nearly 100% coverage [41]. The LISA register has 95% completeness of occupation data and > 98% completeness of education data, and selection bias is minimized, as participation in Swedish government-administered registers such as LISA is compulsory [42]. Similarly, the SKaPa has demonstrated satisfactory reliability and accuracy for dental caries as dft/DFT [43]. However, as the variable e = extracted/M = missing has been shown to be unreliable, it was not used in this study [43]. Regrettably, few private general dental clinics are affiliated with the SKaPa, which explains why SkaPa data were available for only 88.6% of the cohort.

The completeness of treatment data in the studied region is likely unparalleled, as all publicly funded specialist orthodontic care is exclusively provided by the public health dental services. In contrast, regions where publicly funded specialist orthodontic treatment is delivered by multiple organizations would face greater challenges in data retrieval. However, there may be a slight under-registration of orthodontic treatment starts due to clinicians occasionally failing to update the registries. If such under-registration exists, it is likely to be evenly distributed across sociodemographic groups.

Finally, the investigated sociodemographic variables were adjusted for confounders based on DAGitty analyses, strengthening the reliability of the results.

A limitation of this study is that it focuses on data from a single Swedish region. There may be regional disparities in access to specialist orthodontic care, influenced by differences in patient selection criteria and variations in workforce availability. While some regions face staff shortages, others do not. However, investigating interregional differences falls outside the scope of this study but would be a valuable area for future research.

Another limitation of our study is that we were unable to measure malocclusion and orthodontic treatment needs. Instead, we assumed that the distribution of orthodontic treatment need was uniform across socioeconomic, ethnic, and demographic groups, consistent with previous studies [44].

Furthermore, we only measured specialist orthodontic care. To a lesser extent, some removable and interceptive orthodontic appliances are handled at general dentistry clinics. This treatment data was not included in the current study.

## Conclusions

The study suggests that sociodemographic factors influenced the distribution of publicly funded orthodontic care in Region Östergötland, Sweden. Males, individuals born outside the country, those with foreign born parents, and those whose parents had lower education level were less likely to receive treatment compared to their counterparts. However, it remains unclear whether this skewed distribution is driven by differences in treatment demand or other underlying factors.

## Supplementary Information

Supplementary Material 1.

## Data Availability

Raw data and programming code are available from the corresponding author EG upon reasonable request and with the permission of SKAPA, Statistics Sweden and the Swedish Ethical Review Authority. Restrictions apply.

## Abbreviations

DAG: Directed Acyclic Graph
DEGURBA: Eurostat’s Degree of Urbanization
dft: Decayed, filled teeth (primary)
DFT: Decayed, Filled Teeth (permanent)
LISA: Longitudinal Integrated Database for Labor Market and Health Insurance Studies
MGR: Multi Generation Register
PIN: Personal identification number
SCB: Statistics Sweden
TPR: Total Population Register
SKaPa: Swedish Quality Registry for Caries and Periodontal Disease
