# Supplementary material for: Equality of specialist orthodontic care for adolescents in the Swedish public dental service: a cohort study
Source: BMC Oral Health. 2025 May 28;25:841. doi: 10.1186/s12903-025-06220-x (PMC12121215; doi:10.1186/s12903-025-06220-x)
Supplement: Supplementary file 1 — Supplementary Material 1. [file 12903_2025_6220_MOESM1_ESM.docx]

**Supplementary table 1.** Variables, data sources, and data cleaning.

|  | **Data source** | **Variable name in the registry** | **Data cleaning** |
| --- | --- | --- | --- |
| **Primary exposure – Sociodemographic variables** | | |  |
| Sex | SCB/TPR | Kon | - |
| Year of birth | SCB/TPR | FodelseAr | - |
| Country of birth | SCB/TPR | FodGrEg4 | Country of birth, grouped by “EU 28” subdivisions. Dichotomized in our study as foreign born or Swedish born. |
| Linkage to parents/adoptive parents | SCB/MGR | - | Parental data were collected from the biological mother and father, or, if applicable, replaced by adoptive parents. For each parental variable, if the cohort participant had an adoptive parent, the variable was gathered from the adoptive parent and not the biological parent. If linkage to parents was missing, only sociodemographic variables of cohort participants and not of parents of those cohort participants was analysed. |
| Parental country of birth | SCB/TPR | FodGrEg4 | Country of birth, grouped by “EU 28” subdivisions. Dichotomized as foreign born or Swedish born. |
| Parental DEGURBA† | SCB/LISA | Kommun | Degree of urbanization where parents lived at child aged 10 years. Municipality registered as “Kommun”. Each “Kommun” classified in type of DEGURBA= degree of urbanization by the SCB “kn_kopplingar” 2023. Variable “Kommun” measured at year of cohort participant aged 10 years. Mother and father separately. |
| Parental living arrangement ‡ | SCB/LISA | FamId | Each family in SCB is assigned a family ID ("FamId"). Regardless of marital status, parents who live at the same address and have shared children ("gemensamma barn") are given the same family ID. Parental living arrangements was classified as parents living together or separately based on whether the mother and father shared the same family ID when the cohort participant was 10 years old. |
| Parental educational level ‡ | SCB/LISA | Sun2000Niva_Old | Parental highest grade completed at child aged 10 years. The Sun2000Niva_Old aggregates educational level in 7 levels. For our analysis educational level was further condensed into three levels;  Primary/lower secondary school – Sun2000Niva_Old level 1 & 2  Upper secondary school – Sun2000Niva_Old level 3 & 4  University/college education – Sun2000Niva_Old level 5, 6 & 7 |
| Parental social welfare § | SCB/LISA | SocBidrFam | Parental social welfare was classified as “yes” if one or both parents receiving social welfare at any time during the cohort participant's childhood (when aged 0–10 years). Otherwise, parental social welfare was classified as “no”. |
| Parental unemployment ¶ | SCB/LISA | ALosDag | In the SCB registry, the number of unemployment days per calendar year is registered. In our study, parental unemployment was defined as “yes” if the parent had been unemployed for six months or more at any time during the cohort participant's childhood (when aged 0–10 years). Otherwise, parental unemployment was classified as” no”. |
| Parental income # | SCB/LISA | DispInkeKe04 | Net yearly income: The mean income of both parents, adjusted for the number of family members, and categorized into quintiles.  Income was assessed for the year in which the cohort participant was 10 years old. If data for this specific year were unavailable, values were substituted with data from either (1) the following year or (2) the preceding year. If income data were missing for the designated year and both adjacent years, the data were considered missing.  If income data were available for only one parent, the reported mean income was based solely on the available parent's income. |
| **Secondary Exposure - Dental Health variables** | | |  |
| dft primary at age 10 | SKaPa | dft | Data from 2010 (when the registry started) up until 2020 was available and included. Data available for participants registered at SkaPa affiliated clinics. Decayed, filled primary teeth (dft) at age 10. Exfoliation of primary teeth did not erase the occurrence of decay. If data for this specific year were unavailable, values were substituted with data from either (1) the following year or (2) the preceding year. If data were missing for the designated year and both adjacent years, the data were considered missing. |
| DFT permanent at age 14 | SKaPa | DFT | Data from 2010 (when the registry started) up until 2020 was available and included. Data available for participants registered at SkaPa affiliated clinics. Decayed, filled permanent teeth (DFT) age 14 were used. If data for this specific year were unavailable, values were substituted with data from either (1) the following year or (2) the preceding year. If data were missing for the designated year and both adjacent years, the data were considered missing. |
| **Primary Outcome - Initiation of Specialist Orthodontic treatment** | | |  |
| Start of specialist orthodontic treatment | Dental journal records: Edward 32, Pro Curis Inc | 1. pab_beh 2. If missing, Avt_påb OR Fa_påb OR FAST OR Bet barn OR Damon OR Q OR DAM OR Clear | If more than one registrations of treatment start and treatment finish were registered, data from the first treatment episode with fixed appliances was included in further analyses. The variable names in the registry represent different available treatment registrations (in Swedish). |
| **Secondary Outcome - Type and length of Specialist Orthodontic treatment** | | | |
| Type of specialist orthodontic treatment | Dental journal records: Edward 32, Pro Curis Inc | 1. Removable: Avt_påb OR AVTAGB OR Avtagbar 2. Fixed: Fa_påb OR FAST OR Bet barn OR Damon OR Q OR DAM OR Clear | Grouped into removable appliances only or fixed appliances (including those treated with removable and fixed).  The variable names in the registry represent different available treatment registrations (in Swedish). |
| Length of specialist orthodontic treatment | Dental journal records: Edward 32, Pro Curis Inc | 1. pab_beh 2. If missing, Avt_påb OR Fa_påb OR FAST OR Bet barn OR Damon OR Q OR DAM OR Clear 3. Avslutad beh 4. If missing; RET OR Ret 1 enb OR Ret 1enb OR Ret_1 OR Ret_2 OR RET1 OR RET2 OR, 5. if all previous missing: färdigbeh OR AVSL | Difference in months between date ot treatment start (primary outcome) and treatment finish.  If more than one registrations of treatment start and treatment finish were registered, data from the first treatment episode with fixed appliances was included in further analyses.  The variable names in the registry represent different available treatment registrations (in Swedish). |
|  |  |  |  |

**Abbreviations**

DEGURBA Eurostat’s Degree of Urbanization

dft Decayed, filled teeth (primary)

DFT Decayed, Filled Teeth (permanent)

LISA Longitudinal Integrated Database for Labor Market and Health Insurance Studies

MGR Multi Generation Register

PIN Personal identification number

SCB Statistics Sweden

TPR Total Population Register

SKaPa Swedish Quality Registry for Caries and Periodontal Disease
